# *Gastrodia kachinensis* (Orchidaceae), a new species from Myanmar

**DOI:** 10.3897/phytokeys.94.21348

**Published:** 2018-01-29

**Authors:** Ye Lwin Aung, Xiaohua Jin

**Affiliations:** 1 State Key Laboratory of Systematic and Evolutionary Botany, Institute of Botany, Chinese Academy of Sciences, Beijing 100093, China; 2 Southeast Asia Biodiversity Research Institute, Chinese Academy of Sciences, Yezin, Nay Pyi Taw 05282, Myanmar

**Keywords:** Gastrodieae, Kachin, key, montane forest, taxonomy

## Abstract

*Gastrodia kachinensis*, a new species of Orchidaceae, is described and illustrated from Putao, Kachin State, Myanmar. It is morphologically similar to *G. gracilis*, presumably its nearest relative, but can be easily distinguished from the latter by having perianth tube with punctate outer surface, verrucose outer surface of sepal lobe, orbicular petals, ovate-elliptic lip with truncate apex and auriculate-clawed base, glabrous lip apex with a pair of twin protuberance-like lamellae and column with a pair of blade-like lateral wings and acute stelidia at apex. Identification key and colour photographs are provided. A preliminary risk-of-extinction assessment, according to the IUCN Red List Categories and Criteria, is given for the new species.

## Introduction

*Gastrodia* R. Brown (1810: 330) (Orchidaceae, Epidendroideae, Gastrodieae) is composed of approximately 90 species, widespread from northeast India through the eastern Himalayas and southern China to Japan and eastern Siberia, southwards to Malaysia and Australia, eastwards to the Pacific Islands as far as Samoa and westwards to Madagascar, Mascarene Islands and tropical Africa (Pridgeon et al. 2005, Chen et al. 2009, Cribb et al. 2010, The Plant List 2013, Chase et al. 2015, Huang et al. 2015, Hsu et al. 2016). It is characterised by a fleshy tuber or coralloid underground stem, absence of leaves, union of sepals and petals and two mealy pollinia with/without caudicles (Dressler 1993, Seidenfaden and Wood 1995, Leou 2000, Chung and Hsu 2006). Although there is no record of *Gastrodia* in the checklist of Myanmar (Kress et al. 2003), two species of *Gastrodia* were newly recorded in Myanmar recently (Kurzweil and Lwin 2014, Jin and Kyaw 2017). During fieldwork in Putao, Kachin State, Northern Myanmar, in May 2017, a new species of *Gastrodia* was discovered and is described below.

## Material and methods

All measurements of the new species here described, i.e. *Gastrodia kachinensis*, were taken from dried herbarium specimens and field notes. In the description, length and width are represented as length × width. About five living plants and four dried specimens of the new species were examined. In addition, all specimens of *Gastrodia* kept in the Herbarium of Myanmar Forest Department, KUN and PE were examined. Morphological characters of the new species, *Gastrodia gracilis* Blume and other related species, were based on dried herbarium specimens deposited at the Chinese National Herbarium (PE) and on the high resolution photographs of live plants provided by Tian-Chuan Hsu and Xiao-hua Jin.

## Taxonomic treatment

### Gastrodia kachinensis

Taxon classificationPlantaeAsparagalesOrchidaceae

X.H.Jin & L.A.Ye

urn:lsid:ipni.org:names:77175482-1

Figures 1 , 2 , 3

#### Diagnosis.

*Gastrodia kachinensis* is similar to *G. gracilis*, but it can be easily distinguished from the latter by having perianth tube with punctate outer surface, verrucose outer surface of sepal lobe, orbicular petals, ovate-elliptic lip with truncate apex and auriculate-clawed base, glabrous lip with a pair of twin protuberance-like lamellae only at apex and column with a pair of blade-like lateral wings and acute stelidia at apex.

#### Type.

MYANMAR. Kachin State: Putao Township, Hponkanrazi Wildlife Sanctuary, subtropical, evergreen, broad-leaved forest, 1400 m in elevation, 19 May 2017, Xiaohua Jin et al, *PT -6897* (Putao expedition team 6897) (Holotype, PE!).

#### Description.

Terrestrial, fully mycoheterotrophic, leafless herb. Rhizome tuberous, vertical, subterete, 10–14 cm long, ca. 1 cm thick, greyish brown, covered with membranous scales. Stem erect and slender, ca. 30–40 cm long, 0.6 cm thick, distantly noded and sheathed. Raceme laxly 8–10-flowered, peduncle 10–30 cm long, ca. 0.5 cm in diameter, floral bracts minute, erect, lanceolate-ovate, dark brown, acute apex, 6 × 1.5 mm. Pedicel and ovary ca. 1 cm long, pedicel slightly curved, ovary ca. 2.5 mm in diameter. Flowers urceolate, resupinate, bending downwards, dark yellowish brown, ca. 1.3 cm long, 0.8 cm in diameter; sepals connate, forming a tube, tubular part ca. 1 cm long, outer surface punctate, trilobed at apex, sepal lobes ovate, 0.3 × 0.3 cm, outer surfaces verrucose; petals attached to sepal tube, orbicular, 0.2 × 0.2 cm; lip adnate to column foot, orange-yellow towards apex and pale greenish white along each lateral margin, ovate-elliptic, 0.7 × 0.4 cm, margin entire, apex truncate, base auriculate-clawed, two light yellow sub-globose calli at claw, adaxial surface of lip glabrous, with a pair of twin protuberance-like lamellae only at apex, lamellae ca. 1 mm tall; column straight, as long as lip, ca. 0.6 cm long excluding column foot, with a pair of blade-like lateral wings towards apex, stelidia acute at apex; column foot distinct; stigma distinct, near base of column, rounded, ca. 1.5 mm in diameter.

**Figure 1.**
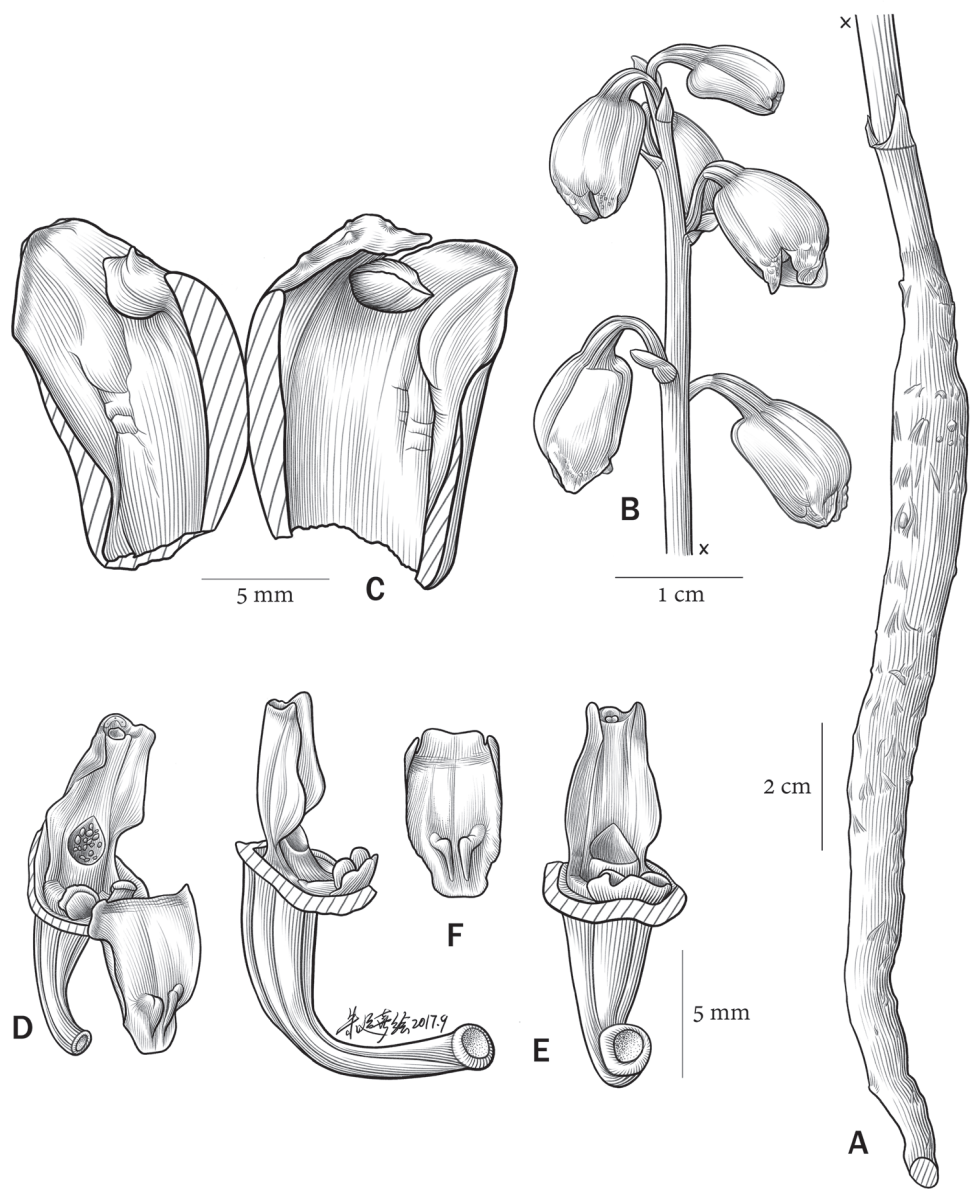
*Gastrodia kachinensis* X.H.Jin & L.A.Ye. **A** Rhizome **B** Inflorescence **C** Longitudinal section of sepal tube, showing two petals **D** Front view of column and lip, showing the stigma area with sectile pollinia **E** Lateral view of column and hypochile, showing a pair of sub-globose calli at lip hypochile **F** Lip epichile, showing a pair of twin protuberance-like lamellae at its apex. Illustration by Yunxi Zhu.

**Figure 2.**
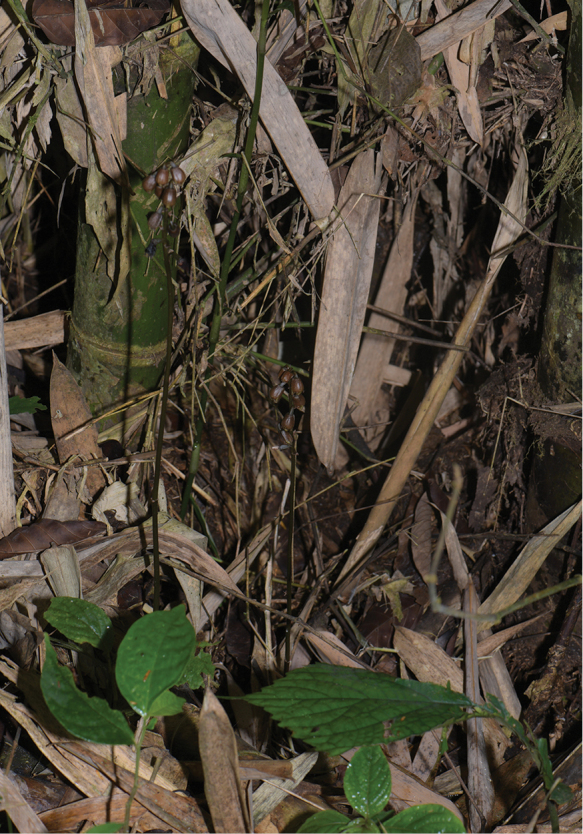
Habit of *Gastrodia kachinensis*. Photographed by X.H. Jin.

**Figure 3.**
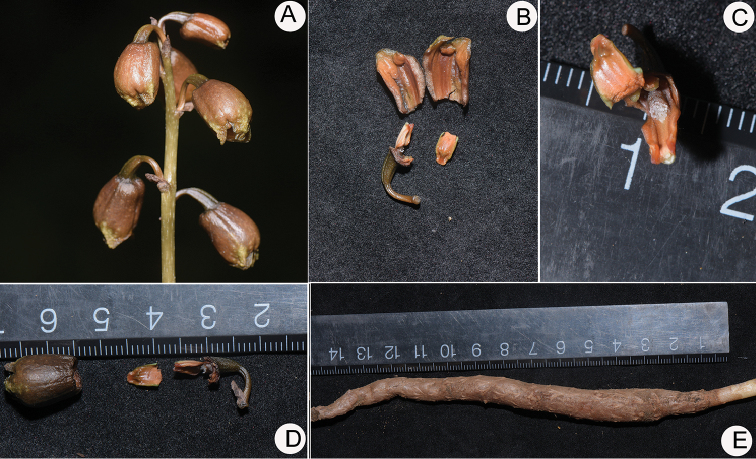
*Gastrodia kachinensis* X.H.Jin & L.A.Ye. **A** Inflorescence **B** Longitudinal section of sepal tube, showing two petals **C** Front view of column and lip, showing the stigma area with sectile pollinia **D** Sepal tube, lip, lateral view of column and the base of lip, showing a pair of sub-globose calli at lip hypochile **E** Rhizome. Photographed by X.H. Jin.

#### Etymology.

The new species is named after Kachin State, the northernmost state of Myanmar in which it was discovered in a vast area of primitive montane forest.

#### Distribution and habitat.

*Gastrodia kachinensis* is a terrestrial mycoheterotrophic species that grows in broad-leaved, evergreen forest at 1400 m in elevation. *Gastrodia kachinensis* is only known from the type locality.

#### Conservation status.

**Endangered (EN).** *Gastrodia kachinensis* was collected in the lowland forest of Hponkanrazi Wildlife Sanctuary, Putao, Northern Myanmar. Until now, only one population, consisting of ca. 10 individuals, has been discovered in the reserve (2704 km^2^). As the lowland forest is under direct threat from slash-and-burn agriculture, the species is here assigned a status of Endangered (EN) according to the guidelines for using the IUCN Red List Categories and Criteria (IUCN Standards and Petitions Subcommittee 2017).

#### Key to *Gastrodia kachinensis* and *G. gracilis*

**Table:** 

1	Perianth tube outer surface punctate; sepal lobes outer surface verrucose; petals orbicular; lip ovate-elliptic, margin entire, apex truncate, base auriculate-clawed, adaxial surface glabrous, with a pair of twin protuberance-like lamellae only at apex; column with a pair of blade-like lateral wings, stelidia acute at apex	***Gastrodia kachinensis***
–	Perianth tube outer surface smooth; sepal lobes outer surface smooth; petals ovate; lip ovate-triangular, margin undulate, apex obtuse, base truncate-clawed, adaxial surface tomentose, with a pair of longitudinal lamellae which are distinctly crested only at apex; column with a pair of semilunar lateral wings, stelidia acuminate at apex	***Gastrodia gracilis***

## Discussion

*Gastrodia kachinensis* and *G. gracilis* are very similar in their vegetative as well as floral morphology. However, *Gastrodia kachinensis* can be easily distinguished from *Gastrodia gracilis* by having perianth tube with punctate outer surface, verrucose outer surface of sepal lobe, orbicular petals, ovate-elliptic lip which is orange-yellow towards apex and pale greenish white along each lateral margin and is composed of entire margin, truncate apex, auriculate-clawed base and glabrous adaxial surface with a pair of twin protuberance-like lamellae only at apex and column with a pair of blade-like lateral wings and acute stelidia at apex (Table 1). *Gastrodia kachinensis* grow at 1400 m in elevation and *G. gracilis* at ca. 600–1500 m in elevation (Chen et al. 2009). In addition, *Gastrodia longitubularis* Q.W. Meng, X.Q. Song & Y.B. Luo and *G. huapingensis* X.Y. Huang, A.Q. Hu & Yan Liu might also be the allied species of *Gastrodia kachinensis* (Meng et al. 2007, Huang et al. 2015), but *G. kachinensis* can be easily distinguished from the former two species (Table 1).

**Table 1.** Morphological comparison of diagnostic features of *Gastrodia kachinensis* and its related species (Meng et al. 2007, Chen et al. 2009, Huang et al. 2015).

Character	*G. kachinensis*	*G. gracilis*	*G. longitubularis*	*G. huapingensis*
Flower position	bending downwards	nodding	horizontal or slightly bending	pointing slightly downwards
Flower colour	dark yellowish brown	dull brownish	grey-brownish	greyish brown
Perianth tube	punctate, urceolate, outer surface of sepal lobe verrucose, petals orbicular	glabrous, ventricose, outer surface of sepal lobe glabrous, petals ovate	glabrous, slender, outer surface of sepal lobe glabrous, petals ovate or sub-rotundate	glabrous, bell-shaped, outer surface of sepal lobe glabrous, petals ovate
Labellum	orange-yellow towards apex and pale greenish white along each lateral margin, ovate-elliptic, apex truncate, base auriculate-clawed, adaxial surface of lip glabrous, with a pair of twin protuberance-like lamellae only at apex	red or orange-red, ovate-triangluar, apex obtuse, base truncate-clawed, adaxial surface of lip tomentose, with a pair of longitudinal lamellae which are distinctly crested only at apex	red or orange-red, ovate or cordate, apex cuspidate, base rounded-clawed, adaxial surface of lip longitudinally 3-5-grooved, with a pair of longitudinal lamellae near apex	pale yellowish brown, ovate, apex truncate, base obtuse-clawed, adaxial surface of lip with 5 longitudinally ridges, of which central two are much longer and more prominent
Column	As long as lip, with a pair of blade-like lateral wings and acute stelidia at apex	As long as lip, with a pair of semilunar lateral wings and acuminate stelidia at apex	Longer than lip, laterally winged from middle to apex, stelidia acute-triangular at apex	Dimorphic column; extended in some flowers, whereas incurved in some flowers

## Supplementary Material

XML Treatment for Gastrodia kachinensis
